# Genomic and phylogenetic evidence of VIPER retrotransposon domestication in trypanosomatids

**DOI:** 10.1590/0074-02760160224

**Published:** 2016-11-16

**Authors:** Adriana Ludwig, Marco Aurelio Krieger

**Affiliations:** 1 Fundação Oswaldo Cruz, Instituto Carlos Chagas, Laboratório de Genômica Funcional, Curitiba, PR, Brasil; 2 Instituto de Biologia Molecular do Paraná, Curitiba, PR, Brasil

**Keywords:** transposable element, trypanosomatids, domestication, reverse transcriptase, evolution

## Abstract

Transposable elements are important residents of eukaryotic genomes and eventually the host can domesticate them to serve cellular functions. We reported here a possible domestication event of the vestigial interposed retroelement (VIPER) in trypanosomatids. We found a large gene in a syntenic location in *Leishmania braziliensis*, *L. panamensis*, *Leptomanas pyrrhocoris*, and *Crithidia fasciculata whose* products share similarity in the C-terminal portion with the third protein of VIPER. No remnants of other VIPER regions surrounding the gene sequence were found. We hypothesise that the domestication event occurred more than 50 mya and the conservation of this gene suggests it might perform some function in the host species.

Transposable elements (TEs) are distinct groups of repetitive DNA sequences that have the ability to move to new sites in genomes. They can be classified into two major classes, retrotransposons (class I) or DNA transposons (class II), based on the transposition mechanism, via RNA or DNA intermediates, respectively (Finnegan 1989). They are an important source of genetic variation in eukaryotes and have a significant role in the structure, function, and evolution of genomes. TE genes have been recruited by host genomes during evolution to perform new cellular functions, a process called domestication. Some important examples are the *rag1/rag2* genes involved in V(D)J recombination, descended from *Transib* DNA transposase and *Fv1*, a *gag* retroviral gene that can protect mouse cells against exogenous retroviruses infections (Best et al. 1996, Volff 2006, Alzohairy et al. 2013).

Trypanosomatids are obligatory protozoan parasites, some of which are etiological agents of neglected diseases in humans (Simpson et al. 2006, Lukes et al. 2014). *Trypanosoma cruzi* and *T. brucei* genome sequences revealed that, although rich in repetitive sequences, only 2-5% of their genomes are derived from retrotransposons (El-Sayed et al. 2005).

Vestigial interposed retroelement (VIPER) is a tyrosine recombinase (YR) retrotransposon from *T. cruzi,* the causative agent of Chagas disease (Lorenzi et al. 2006). The VIPER consensus sequence found on Repbase (Jurka et al. 2005) is shown in Fig. 1A. It encodes three open reading frames (ORFs). The first ORF is predicted to encode a Gag-like protein (446 aa). Downstream this ORF, there are two overlapping genes, encoding a YR protein (335 aa) and a protein with reverse transcriptase (RH) and RNAseH (RH) domains (942 aa), respectively. Alternative start sites for the third gene were also found in some VIPER copies in *T. cruzi*, creating a shorter third ORF that does not overlap with ORF2 (Ludwig et al., unpublished observations). VIPER and short related sequences were also found in *T. brucei* and *T. vivax* genomes (Lorenzi et al. 2006) and remnants of this element were found in *T. rangeli* (Stoco et al. 2014).

**Fig. 1:**
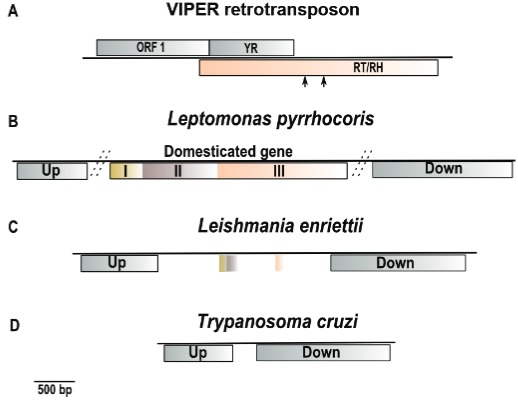
: representation of the vestigial interposed retroelement (VIPER) retrotransposon, the domesticated gene, and the syntenic regions. (A) Schematic representation of the VIPER retrotransposon consensus sequence from *Trypanosoma cruzi*. It has 4390 bp, containing three genes, ORF1 from 169-1509, YR gene from 1510-2517 and RT/RH gene from 1386-4214. Arrows indicate alternative start sites for the third gene; (B) schematic representation of the *Leishmania pyrrhocoris* domesticated gene and its genomic context. The distinct gene regions I, II, and III are indicated with different box colours. The region III is homologous to the C-terminal portion of the third VIPER protein. Up and Down boxes on B, C, and D represent orthologous genes that are upstream and downstream the domesticated gene. The upstream gene has approximately 900 bp and is annotated for some species as “Zn-finger in Ran binding protein and others”. The downstream gene has approximately 1700 bp and is annotated as “hypothetical protein, conserved”. The TriTrypDB gene identification (ID) of these genes for each species can be found in Supplementary Table I. Dotted bars in the intergenic regions represent scale discontinuity; (C) schematic representation of the *L. entiettii* syntenic region showing an intergenic region with remnants of the domesticated gene; (D) schematic representation of the *T. cruzi* syntenic region showing a short intergenic region without any evidence of the domesticated gene.

More than 20 trypanosomatid genome sequences were published or are in progress and are available at TriTrypDB, an important resource for studying TEs in these parasites. Our data shows the presence of VIPER retrotransposons or remnants in several species including *Angomonas deanei*, *Crithidia fasciculata,* and the *Trypanosoma* species (Ludwig et al., unpublished observations). In the course of our studies, we found an interesting gene that suggests VIPER domestication, which is described below.

Using blastP and TblastN searches, the products of VIPER genes 1 and 2 did not show any significant hit (e-value cutoff of 1e^-10^) in *Leishmania* species. Nonetheless, using the third protein alone as query, significant hits were found in *L. panamensis* (5 hits) and two strains of *L. braziliensis* (4 hits). Most hits presented alignments with stop codons and frameshifts, probably corresponding to remnants of VIPER insertions. However, the best hit for these species corresponded to genomic regions of the annotated genes LBRM2903_330043600 and LbrM.33.3490 in *L. braziliensis* and LPAL13_330041800 in *L. panamensis,* assigned as “unspecified product” or “hypothetical protein”. These three genes are clearly orthologous and are found in syntenic locations. Table I shows the information concerning the size and location of these orthologues in the different species and Table II shows the percent of amino acid identity and similarity found between the orthologues. The protein sequences from *L. braziliensis* strains are 100% identical to each other and present 98% identity with the *L. panamensis* protein*.* The alignment of these three proteins with the third VIPER protein extends from the middle to the C-terminus end with approximately 40% similarity. This means that VIPER and this gene greatly differ in their N-terminal portion. Upon analysing the intergenic flanking regions, we were not able to find any evidence of other VIPER regions.

**TABLE I:** Information of the domesticated gene from different species

Strain	Gene ID	Position in chromosome	bp	aa
*Leishmania panamensis* L13	LPAL13_330041800	LpaL13_33:1,371,044 -1,373,989	2946	981
*Leishmania brazilensis* M2903	LBRM2903_330043600	LbrM2903_33:1,546,101-1,549,046	2946	981
*Leishmania brazilensis* M2904	LbrM.33.3490	LbrM.33:1,458,269-1,461,214	2946	981
*Leishmania* sp*.* MAR LEM2494	LMARLEM2494_330040700	LMARLEM2494_33:1,402,644 -1,405,613	2970	989
*Leptomonas pyrrhocoris* H10	LpyrH10_03_5690	LpyrH10_03:1,944,063-1,947,208	2811	937
*Crithidia fasciculata* Cf-C1	Non annotated	CfaC1_21:1263971 -1266907	2937	978

aa: amino acid; bp: base pair; ID: gene identification.

**TABLE II:** Percent amino acid identity (top, right) and similarity (bottom, left) of the domesticated gene among species

		1	2	3	4	5	6
1	LBRM2903_330043600	-	100	98	51	39	40
2	LbrM.33.3490	100	-	98	51	39	40
3	LPAL13_330041800	98	98	-	51	40	39
4	LMARLEM2494_330040700	63	63	63	-	40	39
5	LpyrH10_03_5690	54	54	54	54	-	49
6	*Crithidia*_gene	54	54	53	53	61	-

As indicated on TriTrypDB, the LBRM2903_330043600, LbrM.33.3490, and LPAL13_330041800 genes also have orthologues in syntenic regions in *Leptomonas pyrrhocoris* (LpyrH10_03_5690) and *Leishmania* sp. MAR LEM2494, (LMARLEM2494_330040700). Because of the close proximity of *L. pyrrhocoris* to *L. seymouri* and *C. fasciculata*, we performed searches using TblastN (e-value cutoff of 1e^-10^) for the presence of this gene in both species. In *C. fasciculata,* we found a homologous coding region that was syntenic and not yet annotated (chromosomal coordinates are available in Table I). In *L. seymouri*, we found a related pseudogene in a syntenic location. Fig. 1B shows a representation of the possible domesticated gene and its chromosomal context from *L. pyrrhocoris* as an example. The basic genomic structure for this region is the same as observed in other species.

The amino acid sequences of this gene from the different species and the homologous C-terminal portion of the third VIPER gene were aligned using PSI-Coffee (Floden et al. 2016) (Supplementary data, Figure). Visually, we divided the alignment into three regions that we designated I, II, and III (see Fig. 1B).

Region I extends from position 1 to approximately 140 aa. This short region presents higher identity among sequences than the overall mean identity (99% between *L. braziliensis* and *L. panamensis*; 72% among *Leishmania* sp. MAR LEM2494 and the other *Leishmania* species; 57% among *L. pyrrhocoris* and all *Leishmania* species). This region has no similarity with VIPER or other sequence in the GenBank and lacks protein domains.

Region II is very variable in sequence and size, extending from position 140 to around 430-480 aa. The sequences from the closely related species *L. braziliensis* and *L. panamensis* share 97% identity. However, all other comparisons shared identity around 20%. This region from *Leishmania* sp. MAR LEM2494 has the conserved domain (data from CDD NCBI) Herpes_BLLF1 (e-value 1.22e^-07^) and at the same position, below the threshold (e-value 4.70e-^05^) it matches to the PHA03247 domain (large tegument protein UL36; Provisional). Both domains are present in proteins that compose Herpes virions. *L. pyrrhocoris*, *L. braziliensis, L. panamensis*, and *C. fasciculata* proteins also presented matches with PHA03247, although below the threshold, indicating very low conservation in this domain. The presence of the PHA03247 domain is interesting since viral domains, including the domain under discussion, were found to be part of a special type of TEs, the helitrons (Castañera et al. 2014). However, PHA03247 is the second most frequently found domain in *L. amazonensis* (126 copies) in a less stringent search (Tschoeke et al. 2014). Thus, the actual existence of this domain may be questioned. To determine the origin of this region, we conducted several blast searches, but this region seems to be species-specific. Using the SMART tool (Letunic et al. 2012), we observed that this region contains several low-complexity regions (LCRs) in all species, being highly enriched in the amino acids alanine (A), proline (P), and serine (S). These LCRs, however, are more complex than just single amino acid tandem repeats.

Region III contains approximately 500 aa and is homologous to the C-terminal portion of the third VIPER protein. Although containing only modest similarity to VIPER (around 40%), it is unlikely that evolution has generated a long sequence with such similarity only by chance. The RT and RH domains were not conserved, suggesting that the function of this gene is likely not dependent on these domains, and the new gene function may differ from the original function.

It is still not possible to determine the role of this gene. However, considering its large size (approximately 3 kb) and the conservation of the ORF among several species, we expect this gene encodes a biologically relevant protein since long ORFs rarely occur in random sequences and probably result from selection to remove the stop codons (Li 1999). Moreover, this is a typical scenario, since the function of most TE-derived genes is not well understood so far (Volff 2006).

Our hypothesis that this gene resulted from a TE domestication event is based mainly on the following: (i) the gene apparently contains a single copy and is a chimera of the third VIPER gene and some other sequence, thus, it is clearly a TE-derived sequence; (ii) the gene has been conserved in synteny in some species for several million years, indicating it is playing a role; and (iii) there is no evidence of other VIPER regions surrounding the gene.

To evaluate when the domestication event occurred, we analysed the trypanosomatid genomes from a phylogenetic perspective. A representation of the phylogenetic relationships of trypanosomatids was created based on well-supported phylogenies from several studies (Fig. 2). All species analysed here were included, but they represent only a small number of all trypanosomatids; thus, the evolutionary inferences can be further updated with studies on additional species.

**Fig. 2:**
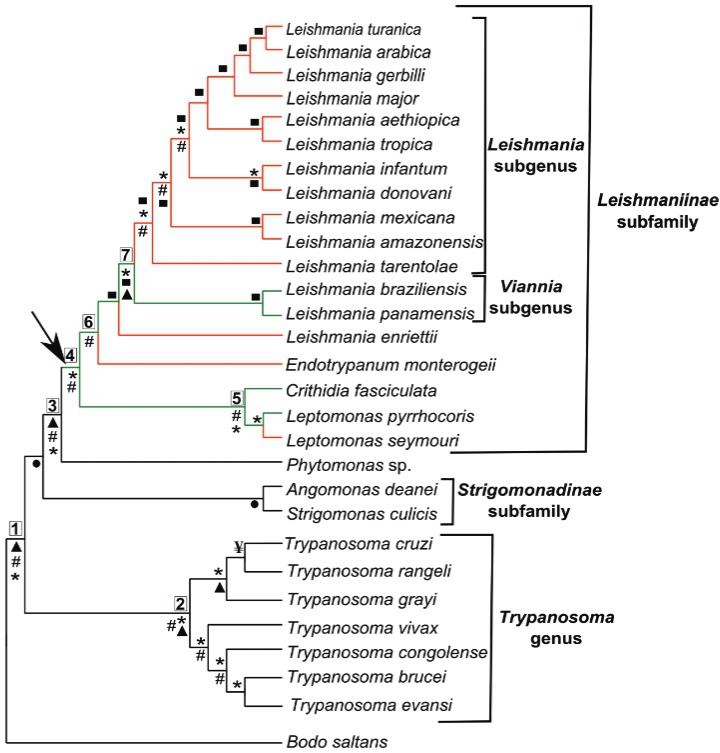
: scheme of phylogenetic relationships among different trypanosomatids employed in this study based on several works. *Bodo saltans* was represented as an outgroup. Green and red branches represent the presence and loss of the domesticated gene, respectively. The black arrow indicates the ancestor where the gene was likely domesticated. Numbers in boxes near some nodes are references to the divergence time estimates in mya (millions years ago), as inferred by Lukes et al. (2014): 1 - 231-283 mya; 2 - 96-105 mya; 3 - 118-170 mya; 4 - 52-96 mya; 5 - 30-63 mya; 6 - 31-65 mya; 7 - 25-54 mya. Marks near nodes indicate the works that support that branching: * - Flegontov et al. (2016); # - Flegontov et al. (2013) and Lukes et al. (2014); ¥ - Stoco et al. (2014); ▲ - Kelly et al. (2014); • - Du et al. (1994), Teixeira et al. (2011) and Votýpka et al. (2012); ▀ - Harkins et al. (2016). Only some relationships from each work were shown.

We then analysed the syntenic chromosomal regions, where the gene is supposed to be present, from the other species. All other *Leishmania* species and *Endotrypanum monterogeii* contain only short sequences that match the domesticated gene to some extent in the syntenic region, showing that the coding capacity was lost in these species. A representation of this genomic region from *L. enriettii* is shown in Fig. 1C. Considering the trypanosomatid phylogeny, we suggest the gene was lost at least four times, in *L. seymmouri*, *E. monterogeii*, *L. enriettii*, and in the ancestor of the *Leishmania* subgenus.

*A. deanei*, *Strigomonas culicis*, and *Phytomonas* sp*.* (isolate EM1) genomes were obtained from the Ensembl Genomes resource (Kersey et al. 2016). *A. deanei* and *S. culicis* contain short contigs, making synteny analysis difficult; however, by applying TblastN (e-value cutoff of 1e^-10^) throughout the genome and querying the sequences from the six species, no significant match was found with the N-terminal portion of the protein. *A. deanei* presented several hits for the C-terminal portion corresponding to copies of the VIPER retrotransposon. In *Phytomonas* sp., the genomic region where the gene is predicted to be present does not contain any evidence of presence or remnants of this gene. The absence of the domesticated gene or its traces in these species suggests that domestication probably occurred in the ancestor of the *Leishmaniinae* subfamily after its separation from the ancestor of the *Phytomonas* genus, estimated to be 118-170 mya, and before the split of *Crithidia* and *Leishmania* clades estimated to be 52-96 mya. The black arrow in Fig. 2 indicates the possible origin of the domesticated gene in the trypanosomatids evolutionary history.

The syntenic genomic regions from all *Trypanosoma* species do not show any evidence of this gene or VIPER insertions, supporting the late origin of this domesticated gene. The *T. cruzi* chromosomal region is shown in Fig. 1D. The other *Trypanosoma* species show the same structure with a very short intergenic region where the domesticated gene should be present.

For all species, in addition to searching for the domesticated gene in the syntenic region, we performed searches throughout the genome, discarding the possibility that the location of the gene was altered during evolution.

We presented a possible event of VIPER retrotransposon gene domestication that occurred more than 50 mya in trypanosomatids. *L. pyrrhocoris* and *C. fasciculata* are monoxenous species that infect insects, and *Leishmania* are dixenous species with alternating hosts (insects and vertebrates) during their life cycles. Considering that the dixenous life style most likely evolved independently from the monoxenous style in the genera *Trypanosoma*, *Leishmania*, and *Phytomonas* (Lukes et al. 2014), we can conclude the domestication event occurred in a monoxenous insect parasite ancestor.

The presence of the gene regions I and II that are apparently not homologous to VIPER suggests it could be a chimeric gene. Chimeric genes are formed during the evolution of genomes through the fusion of different coding regions and may contribute to the evolution of novel functions (Rogers et al. 2009). The origin of regions I and II, however, is not clear. Alternatively, as the third VIPER ORF is about the same size as the domesticated gene, it is possible that the entire domesticated gene was derived from VIPER followed by extreme diversification of the first half.

The high variability found in region II could be explained by the absence of selective constraints in this region combined with the actions of replication slippage and recombination that occur more frequently in low-complexity regions (DePristo et al. 2006). Nonetheless, we cannot rule out the possibility that positive selection is acting due to some role in a genetic conflict. Positive selection can occur for domesticated TEs involved in host defence (Best et al. 1996, Malik & Henikoff 2005). As a region prone to mutations, the appearance of frameshifts and stop codons would easily promote pseudogenisation; therefore, the conservation of the coding capacity implies some degree of functionality and strongly supports domestication.

The loss of the domesticated gene in some species during trypanosomatid evolution suggests that other genes further provided its function or it was no longer necessary. Future studies will help to address the possible function of this gene.

The first example of TE domestication in trypanosomatids showed the involvement of a whole family of short and extinct TEs in regulating gene expression in *Leishmania* species (Bringaud et al. 2007). Here, we provide evidence of an additional domestication event using an *in silico* analysis. Evolutionary inferences were made based on the available data considering the most parsimonious assumption, although we do not discard alternative hypotheses. The addition of more species in the analysis, the completion of on-going genome sequencing projects, and our further experimental functional investigations will certainly help achieve a more comprehensive picture of the role and evolution of this gene. Despite the low diversity and quantity of TEs found in these parasite genomes, the mobile sequences might serve as a good source of evolutionary novelties in trypanosomatids.
