# Supplementary material for: Genomic and phylogenetic evidence of VIPER retrotransposon domestication in trypanosomatids
Source: Mem Inst Oswaldo Cruz. 2016 Nov 16;111(12):765–9. doi: 10.1590/0074-02760160224 (PMC5146736; doi:10.1590/0074-02760160224)
Supplement: Supplementary file 1 [file 0074-0276-mioc-0074-02760160224-suppl01.pdf]

TABLE

TryTripDB gene identification (ID) of the orthologous genes located up and downstream the domesticated gene. The upstream gene is annotated for some species as "Zn-finger in Ran binding protein and others" and the downstream gene is annotated as "hypothetical protein, conserved". Species that contain the domesticated gene are shown in bold. The IDs of orthologous genes from species where the domesticated gene was not found were also provided

Organism	Upstream gene	Downstream gene
***Crithidia fasciculata* strain Cf-Cl**	CfaC1_21: 1262421- 1263227*	CFAC1_210044900
Endotrypanum monterogeii strain LV88	EmoLV88_33: 1359639- 1360553*	EMOLV88_330037700
***Leishmania panamensis* L13**	LPAL13_330041700	LPAL13_330041900
Leishmania aethiopica L147	LAEL147_000701800	LAEL147_000701900
Leishmania arabica strain LEM1108	LarLEM1108_33: 1307955- 1308869*	LARLEM1108_330039800
***Leishmania braziliensis* MHOM/BR/75/M2903**	LBRM2903_330043500	LBRM2903_330043700
***Leishmania braziliensis* MHOM/BR/75/M2904**	LbrM.33: 1456663-1457580*	LbrM.33.3500
Leishmania donovani BPK282A1	Ld33_v01s1: 1435155-1436069	LdBPK_333370.1
Leishmania enriettii strain LEM3045	LENLEM3045_330042100	LENLEM3045_330042200
Leishmania gerbilli strain LEM452	LGELEM452_330042900	LGELEM452_330043000
Leishmania infantum JPCM5	LinJ.33: 1429595-1430509*	LinJ.33.3370
Leishmania major strain LV39c5	LMJLV39_330045400	LMJLV39_330045500
Leishmania major strain SD 75.1	LMJSD75_330044200	LMJSD75_330044300
Leishmania mexicana MHOM/GT/2001/U1103	LmxM.32: 1390571-1391485*	LmxM.32.3220
***Leishmania* sp. MAR LEM2494**	LMARLEM2494_330040600	LMARLEM2494_330040800
Leishmania tarentolae Parrot-TarII	LtaP33.3470	LtaP33.3480
Leishmania tropica L590	LTRL590_330041700	LTRL590_330041800
Leishmania turanica strain LEM423	LTULEM423_330043000	LTULEM423_330043100
***Leptomonas pyrrhocoris* H10**	LpyrH10_03_5680	LpyrH10_03_5700
Leptomonas seymouri ATCC 30220	Lsey_0124_0160	Lsey_0124_0180
Trypanosoma brucei Lister strain 427	Tb427.02.6070	Tb427.02.6080
Trypanosoma brucei TREU927	Tb927.2.6070	Tb927.2.6080
Trypanosoma brucei gambiense DAL972	Tbg.972.2.4240	Tbg.972.2.4250
Trypanosoma congolense IL3000	TcIL3000_2_1690	TcIL3000_2_1700
Trypanosoma cruzi CL Brener Esmeraldo-like	TcCLB.507105.20	TcCLB.507105.30
Trypanosoma cruzi CL Brener Non-Esmeraldo-like	TcCLB.506815.40	TcCLB.506815.50
Trypanosoma cruzi Dm28c	TCDM_07380	TCDM_07379
Trypanosoma cruzi Sylvio X10/1	TCSYLVIO_004016	TCSYLVIO_004017
Trypanosoma cruzi marinkellei strain B7	Tc_MARK_2783	Tc_MARK_2784
Trypanosoma evansi strain STIB 805	TevSTIB805.2.3440	TevSTIB805.2.3450
Trypanosoma grayi ANR4	Tgr.572.1040	Tgr.572.1050
Trypanosoma rangeli SC58[1]	TRSC58_00071	Not found
Trypanosoma vivax Y486	TvY486_0202070	TvY486_0202080

*: non annotated orthologues. The chromosomal position of the open reading frame is indicated; [1]: the downstream gene was not found for this species and the upstream gene is located in a small contig.

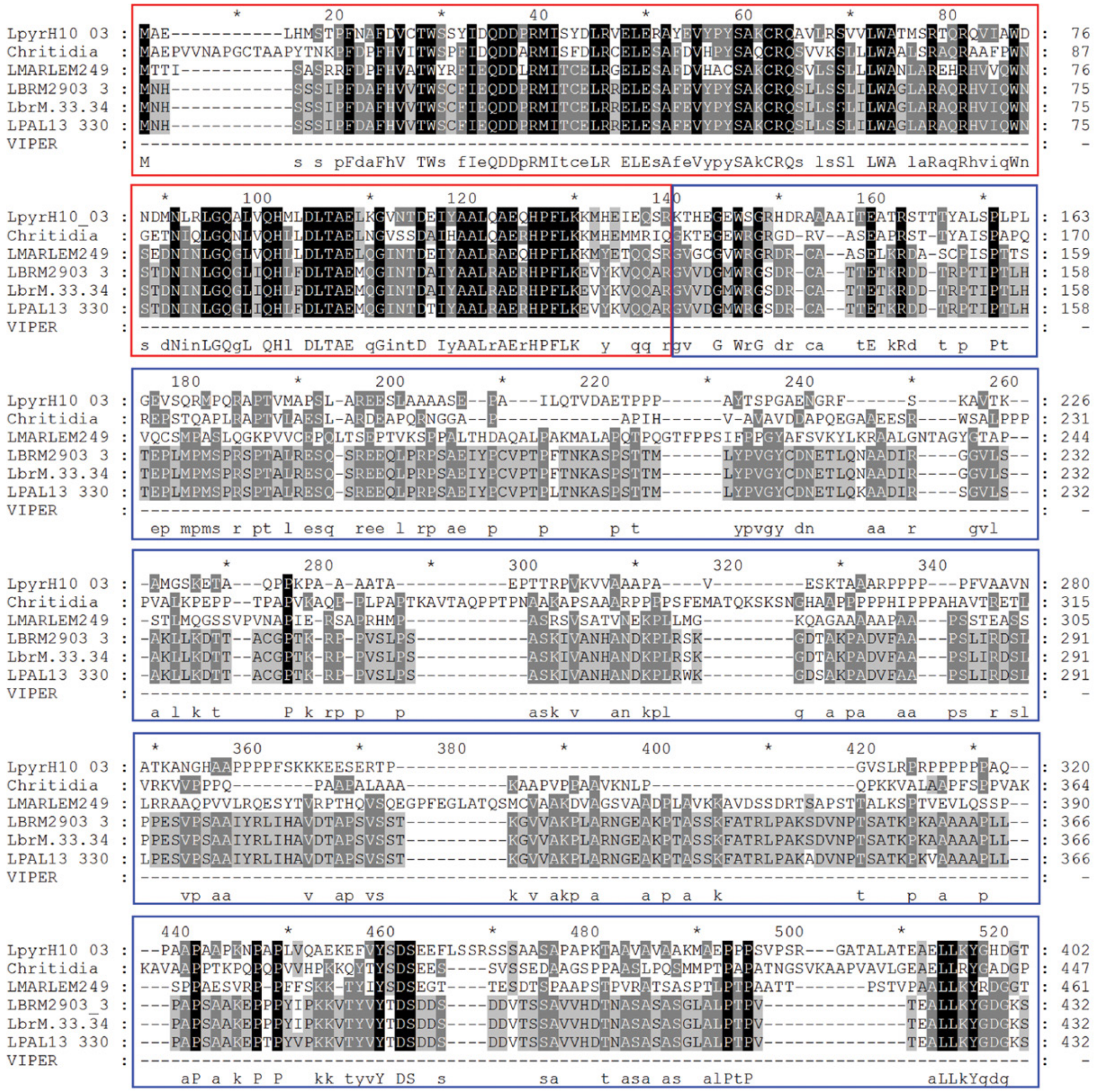
LpyrH10 03
Chritidia
LMARLEM249
LBRM2903 3
LbrM.33.34
LPAL13 330
VIPER

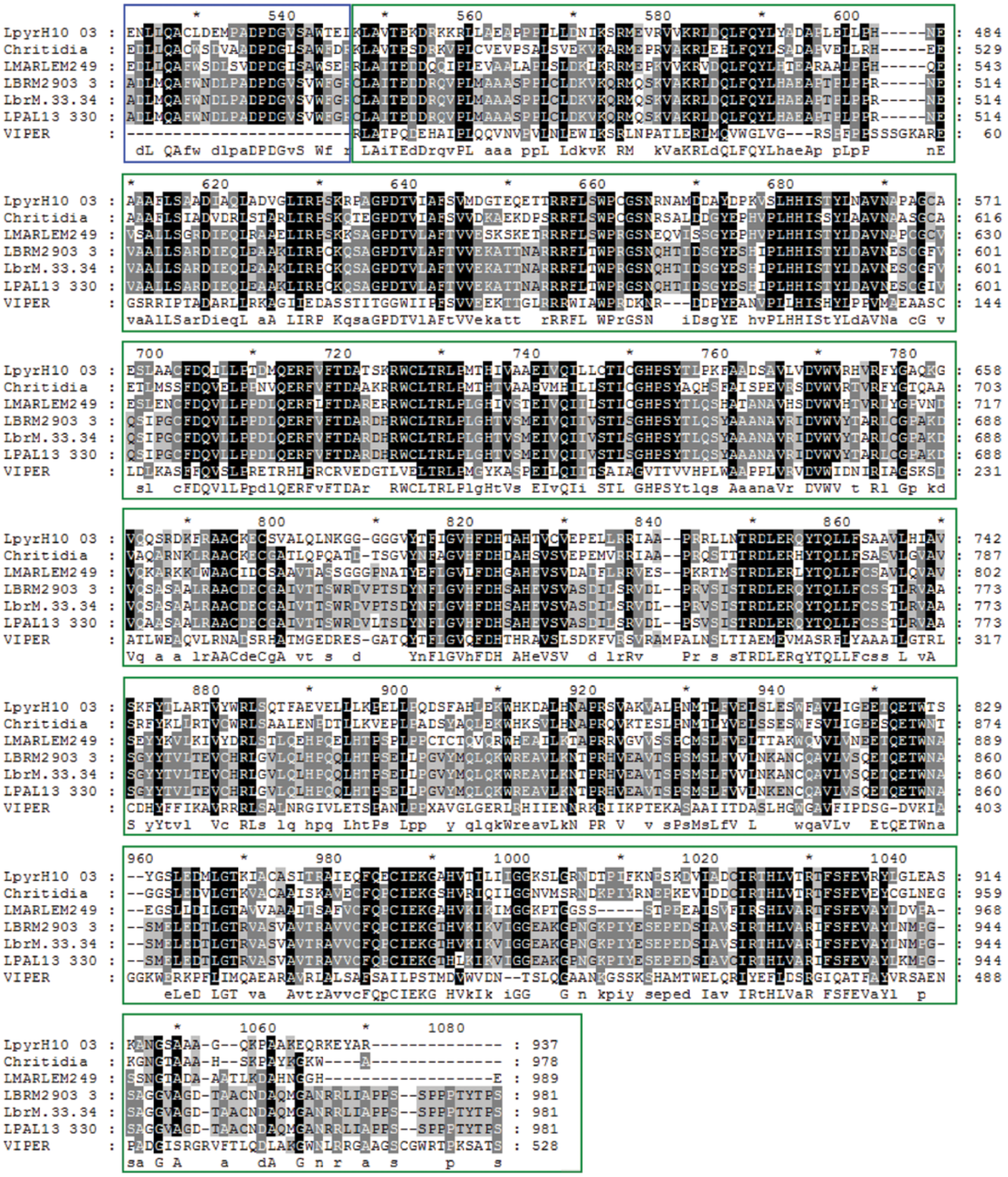

Alignment view of amino acid sequences of the domesticated gene from different species and the C-terminal portion of the third VIPER protein. The distinct regions I, II, and III of the protein are represented by red, blue, and green boxes, respectively. The order of genes from top to bottom is LpyrH10_03_5690, *Crithidia* non-annotated gene, LMARLEM2494_330040700, LBRM2903_330043600, LbrM.33.3490, LPAL13_330041800, and VIPER.
